# Disparities in perinatal outcomes among births to same-sex and different-sex married couples: a cross-sectional study using birth certificate data

**DOI:** 10.1016/j.xfre.2025.04.005

**Published:** 2025-04-23

**Authors:** Kendall A. Lawley, Brittany M. Charlton, Elizabeth Rubin, Paula Amato, Jae Corman

**Affiliations:** a Department of Epidemiology, University of Washington, Seattle, Washington; b Department of Population Medicine, Harvard Medical School and Harvard Pilgrim Health Care Institute, Boston, Massachusetts; c Department of Epidemiology, Harvard T.H. Chan School of Public Health, Boston, Massachusetts; d Division of Reproductive Endocrinology and Infertility, Department of Obstetrics and Gynecology, Oregon Health & Science University, Portland, Oregon; e Folx Health, Boston, Massachusetts

**Keywords:** Perinatal outcomes, health disparities, LGBTQ, preterm birth, macrosomia

## Abstract

**Objective:**

To evaluate differences in birth outcomes for infants born to parents in same-sex versus different-sex marriages.

**Design:**

Cross-sectional study

**Subjects:**

Birth certificate data from Ohio for 318,616 singleton infants born to married parents from 2016 to 2020.

**Exposure:**

We estimated the associations of having a parent in a same-sex marriage (SSM) vs. a different-sex marriage (DSM).

**Main Outcome Measures:**

Preterm birth, low birth weight, macrosomia, small infant for gestational age, large infant for gestational age, and whether these outcomes differed by use of assisted reproductive technologies.

**Results:**

A total of 1,661 singletons were born to married birthing people in SSM. Preterm birth was more common in birthing persons in SSM compared with those in DSM (11.0% vs. 7.6%), and this difference remained significant on multivariate analyses controlling for covariates. Size for gestational age was similar among infants with parents in SSM and DSM. Among those who used assisted reproductive technologies, infants born to parents in SSM had a 1.58 (95% CI: 1.15, 2.12) higher odds of macrosomia compared with those in DSM.

**Conclusion:**

People in SSM may face modestly increased risks for some perinatal outcomes, including preterm birth. Understanding the risks and resiliency among diverse sexual minority populations may provide important insights into the ways in which people in same-sex partnerships achieve healthy perinatal outcomes.

Legal and technological advances are making childbirth more accessible to many same-sex couples, however, we know little about how perinatal outcomes differ between same-sex female couples and different-sex couples. Emerging research has revealed that sexual minority people who give birth experience worse perinatal outcomes than heterosexual people (1, 2). Minority stress theory posits that sexual minority individuals face unique stressors because of marginalization, which can contribute to poor health outcomes (3, 4). Minority stress may play a role in these disparities as it is associated with poor perinatal outcomes, including low birth weight (LBW), preterm birth (PTB), and fetal growth restriction (1). It is also important to note the role of intersectionality in birth outcomes research. Some recent research has shown that Black and Latina sexual minority women experience worse birth outcomes compared with Black and Latina heterosexual women as well as white sexual minority women, suggesting that minority stress related to sexual identity may be additionally compounded by experiences of racism (5). We also know that pregnancies conceived using assisted reproductive technologies (ART) are associated with adverse outcomes, including PTB and LBW (6, 7). As many same-sex couples use ART as a pathway to pregnancy (8), it is important to understand how ART may influence perinatal outcomes in this population. One recent study examining the different pathways to conception between same-sex partnered cisgender women compared with different-sex partnered cisgender women found that same-sex partnered participants were nearly 5 times more likely to use medically assisted reproduction than different-sex partnered participants (9). These findings demonstrate a need to consider the role of ART and other forms of medically assisted reproduction in birthing outcomes for birthing persons in same-sex partnerships.

One barrier to understanding perinatal outcomes for same-sex couples is the paucity of data on sexual orientation in nationally representative data. Methodologically rigorous, large-scale studies of perinatal outcomes with data on sexual orientation are largely absent; prior studies have used nonprobability methods or contain small sample sizes (1). One strategy that has shown to be beneficial in addressing this gap is using vital records in the form of birth certificate data. Most states allow legally married same-sex couples to include both partners’ names on their children’s birth certificates. Thus, same-sex marriages (SSM) identified through birth certificates can be used as a proxy for parent sexual orientation.

A prior study utilizing birth certificate data to compare perinatal outcomes in Massachusetts found no considerable differences between couples in SSM and different-sex marriages (DSM) (10) suggesting that certain protective factors (e.g., higher education, income) can equilibrize perinatal outcomes for SSM and DSM couples, despite minority stress. However, Massachusetts represents a specific social-ecological context; it was one of the first states to legalize SSM and has a mandate requiring commercial health insurance to cover fertility services, although access to these services may differ on the basis of how infertility is defined within insurance policies (11). Another study using birth certificate data from California found that birthing people in same-sex partnerships had worse obstetrical and perinatal outcomes including the need for induced labor, postpartum hemorrhage, and severe morbidity compared with birthing people in different-sex partnerships (2). Similarly, a study using birth certificate data in Louisiana found that birthing persons in same-sex relationships had higher odds of gestational hypertension and gestational diabetes than birthing persons in different-sex relationships, but no differences were found for PTB or LBW (12). These 3 studies provide valuable information on birth outcomes for SSM in the Northern, Western, and Southern United States. To date, no studies have specifically focused on exploring differences in birth outcomes in the context of the Midwestern region of the United States. There is substantial variability in the social and political contexts of different regions in the United States, which can have notable impacts on access to reproductive health services and general acceptance of same-sex couples. This variability makes it challenging to generalize findings across regions, so it is imperative to explore these associations in other settings to examine whether similar patterns of perinatal outcomes may be found for birthing people depending on the sociopolitical climates of the states in which they reside. The differences across these studies suggest that geographic context likely plays a role in birth outcomes disparities between SSM and DSM couples. The protective factors found in the Massachusetts study were previously discussed, but the California and Louisiana studies are not as consistent. Louisiana is a politically conservative state with relatively unfavorable views of SSM (13). California, was also one of the first states to legalize SSM, removed this right in 2008, and reinstated SSM in 2013 (14, 15). The acceptance of SSM in Ohio falls somewhere between the previously studied states (16), but general studies of health and well-being of sexual and gender minority adults in Ohio report relatively high levels of minority stress (17, 18). Adding to this body of work using data from Ohio can help us move toward a more complete understanding of the reproductive health landscape for same-sex couples across the United States.

Based on previous research demonstrating a link between minority stress and poor birth outcome including PTB, LBW, and restricted fetal growth (1) and studies of general minority stress in Ohio (17, 18) the present study uses birth certificate data from Ohio to evaluate perinatal outcomes for couples in SSM and DSM. Using minority stress theory as the basis for this study, we aimed to investigate whether couples in SSM would experience worse perinatal outcomes, with an emphasis on PTB, than couples in DSM.

## Materials and methods

### Study sample

In the present cross-sectional study, data were collected from birth certificates in Ohio for all live births that occurred from 2016 to 2020 (n = 680,831). Births were excluded if the birthing parent was not listed as married on the birth certificate or whose gender was listed as male; no parents were listed as intersex. Analyses were restricted to singleton births with plausible birth weights and a gestational age between 22 and 45 weeks. The final analytical sample consisted of 318,616 live births. This study was approved by both the Ohio Department of Health and the Oregon Health & Science Institutional Review Boards.

### Measures

#### Relationship status

The predictor of interest was whether the birthing parent was in a DSM (i.e., birthing parent was female and other parent was male) or an SSM (i.e., both parents were female).

### Outcome variables

#### Preterm birth

Preterm birth (<37 completed weeks’ gestation) was the primary outcome of interest and was determined on the basis of last menstrual period and ultrasound data; any discrepancies used ultrasound data (19).

#### Decreased fetal growth

Decreased fetal growth was assessed by LBW and if the infant was small for gestational age (SGA). Low birth weight was defined as <2,500 g and was selected to facilitate comparison with other studies, SGA was calculated by first estimating birth weight for gestational age (BWGA) percentile, expressing fetal growth without the effect of delivery timing. We calculated BWGA from the sex-specific birth tables (20). Small for gestational age was defined as birth weight <10th percentile of BWGA and we selected SGA because acute and chronic stressors can slow fetal growth and accelerate timing of delivery (21), thereby increasing risk for lower educational attainment and later adverse health (22).

#### Excessive fetal growth

Excessive fetal growth was assessed by macrosomia and if the infant was large for gestational age (LGA). Macrosomia was defined as >=4,000 g and was selected to facilitate comparison with other studies. Large for gestational age was calculated by first estimating BWGA percentile, expressing fetal growth without the effect of delivery timing. We calculated BWGA from the sex-specific birth tables (20). Large for gestational age was defined as birth weight >90th percentile, and we examined LGA because of the risk for delivery complications (23) and various subsequent outcomes, including childhood obesity (24).

#### Covariates

We examined infant sex along with birthing parent parity and age (<35; 35+). The birthing parent’s educational attainment was categorized as high school or less; some college; bachelor’s degree; master’s or doctoral degree. Because it is important to have conceptual and scientific justification when adjusting for race/ethnicity (25), we regarded race/ethnicity as a marker of exposure to racism and categorized this as non-Hispanic White, Hispanic, non-Hispanic Black, and another non-Hispanic race/ethnicity. Prepregnancy body mass index (BMI) ≥30 was categorized as obese. Given previous research demonstrating that sexual minority women are more likely to have a BMI classified as obese (26), we explored the potential impact of body size by including both BMI and height (<68 inches; 68+ inches) as covariates. Additionally, the birthing parent’s body size, in part, determines their offspring’s birth weight and height and should be considered as a separate predictor from BMI (27, 28). The Special Supplemental Nutrition Program for Women, Infants, and Children (WIC program) was used as a proxy for socioeconomic status. Participants who reported smoking 3 months before pregnancy or during pregnancy were considered current smokers. Finally, the year of birth was included as a covariate in our adjusted models.

#### Use of ART

Birthing parents who used any of the following to conceive their pregnancy on their hospital record were considered to have used ART: in-vitro fertilization (IVF), fertility-enhancing drugs (e.g., gonadotropins, Gonadotropin-releasing hormone agonists or antagonists, or progesterone), gamete intrafallopian transfer, zygote intrafallopian transfer, intracytoplasmic sperm injection, frozen embryo transfer, or donor embryo transfer. These ART methods were selected because they may increase PTB by twofold among singleton births (29) and increase the risk for SGA and LGA neonates (30). Use of intrauterine and intracervical insemination was not categorized as ART, but these participants were still included in the present study. Because fertility-enhancing drugs can be prescribed for these procedures, couples using intrauterine and intracervical insemination could still be classified as using ART when fertility-enhancing drugs were used, but this means that couples who used intrauterine and intracervical insemination without the addition of fertility-enhancing drugs would be categorized as not using ART.

### Statistical analysis

#### Main models

We fit 2 logit models for each outcome (PTB, LBW, SGA, LGA, and macrosomia). We first calculated unadjusted (crude) odds ratios (OR) and then adjusted for covariates (infant sex, parity, birthing parent age, educational attainment, birthing parent race/ethnicity, prepregnancy obesity, height, use of WIC, smoking status, and year of birth).

#### Differences by use of ART

To assess the potential association between ART and our outcome variables, we also calculated estimates that were stratified by ART use. All statistical analyses were performed in R (Version 4.1.0). All significance tests were two-tailed, and a *P*-value <.05 was considered statistically significant.

## Results

Among the eligible 318,616 singleton births to married birthing people, 0.52% (1,661) were to parents in SSM. Compared with those in DSM, a higher proportion of birthing parents in a SSM were nulliparous (54% vs. 35%), were age 35 or older (22% vs. 19%), had a prepregnancy BMI classified as “obese” (38% vs. 26%), used WIC (19% vs. 14%), were current smokers (8.9% vs. 4.8%), and used ART or other fertility treatments (27% vs. 2.9%). A lower proportion of SSM had a bachelor’s degree or higher compared with DSM (46% vs. 51%). A higher proportion were NH Black compared with DSM (9.8% vs. 7.3%). No significant differences were found for height between SSM and DSM (Table 1). All demographic variables had <1% missing data. The prevalence of decreased fetal growth (SGA and LBW) and PTB was higher for SSM, whereas excessive fetal growth (LGA and macrosomia) was similar for SSM and DSM (Table 2). Small for gestational age and LGA had 6.2% missing data, PTB had 4.7% missing data. Low birth weight and macrosomia each had <1% missing data.

**Table 1:** Characteristics of singleton births to same- and different-sex married couples in Ohio, 2016–2020.

Characteristic	Different-Sex, n = 316,955, n (%)	Same-Sex, n = 1,661, n (%)	*P* value^a^
Nulliparous	110,199 (35)	894 (54)	<.001
Age 35+ (y)	60,553 (19)	364 (22)	.004
Education			<.001
HS or less	74,244 (23)	430 (26)	
Some college	81,471 (26)	465 (28)	
Bachelors	97,803 (31)	418 (25)	
Masters/doctorate	63,016 (20)	344 (21)	
Race and ethnicity			<.001
White NH	262,433 (83)	1,393 (84)	
Hispanic	12,717 (4.0)	51 (3.1)	
Black NH	23,180 (7.3)	163 (9.8)	
Another NH	18,366 (5.8)	54 (3.3)	
Obese prepregnancy	83,075 (26)	621 (38)	<.001
Tall (68+ inches)	45,036 (14)	225 (14)	.4
WIC program	44,637 (14)	317 (19)	<.001
Current smoker	15,170 (4.8)	148 (8.9)	<.001
ART or fertility drug	8,279 (2.9)	414 (27)	<.001

ART = assisted reproductive technology; HS = high school; NH = non-Hispanic; WIC = Special Supplemental Nutrition Program for Women, Infants, and Children.

a Two-tailed tests between people in same and different-sex marriages.

**Table 2:** Differences in birth outcomes between those born to same- and different-sex parents.

	SSM (N = 1,661) (%)	DSM (N = 316,955) (%)	Crude OR (95% CI)	Adjusted OR (95% CI)
Preterm birth	11.0	7.6	1.48 (1.26, 1.72)	1.30 (1.11, 1.52)
SGA	9.8	8.0	1.25 (1.06, 1.46)	1.07 (0.90, 1.26)
LBW	5.7	4.1	1.41 (1.14, 1.73)	1.11 (0.89, 1.37)
LGA	8.0	7.7	1.04 (0.87, 1.24)	1.13 (0.94, 1.35)
Macrosomia	9.3	9.3	1.00 (0.84, 1.18)	1.11 (0.93, 1.31)

CI = confidence interval; DSM = different-sex marriage; LBW = low birth weight; LGA = large for gestational age; OR = odds ratio; SGA = small for gestational age; SSM = same-sex marriage.

### All couples

Results from the crude model showed odds were higher of PTB (OR = 1.48, 95% confidence interval [CI] = 1.26, 1.72) and LBW (OR = 1.41, 95% CI = 1.14, 1.73) among infants born to parents in an SSM compared with DSM. There were no differences in other outcomes. Results from the adjusted model showed only higher odds of PTB (OR = 1.30, 95% CI = 1.11, 1.52) among infants born to parents in an SSM. No other differences were observed (Table 2).

### Couples who used ART

After stratifying by ART use, results from the crude model showed the odds of macrosomia (OR = 1.58, 95% CI = 1.15, 2.12) were higher among infants born to parents in an SSM who used ART than those in a DSM who used ART. The adjusted model for those who used ART showed higher odds of LGA (OR = 1.42, 95% CI = 1.00, 1.98) and macrosomia (OR = 1.64, 95% CI = 1.19, 2.22) for infants born to parents in an SSM than a DSM (Table 3).

**Table 3:** Differences in birth outcomes between those born to same- and different-sex parents who used ART.

	SSM (N = 414) (%)	DSM (N = 8,236) (%)	Crude OR (95% CI)	Adjusted OR (95% CI)
PTB	13.0	11.3	1.18 (0.87, 1.57)	1.08 (0.80, 1.45)
SGA	9.9	9.2	1.08 (0.77, 1.49)	1.04 (0.73, 1.44)
LBW	7.0	6.8	1.03 (0.68, 1.48)	0.93 (0.61, 1.35)
LGA	9.7	7.2	1.38 (0.97, 1.91)	1.42 (1.00, 1.98)
Macrosomia	12.3	8.1	1.58 (1.15, 2.12)	1.64 (1.19, 2.22)

CI = confidence interval; DSM = different-sex marriage; LBW = low birth weight; LGA = large for gestational age; OR = odds ratio; SGA = small for gestational age; SSM = same-sex marriage.

### Couples who did not use ART

Among those who did not use ART, results from the crude model showed infants whose parents were in an SSM had higher odds of PTB (OR = 1.37, 95% CI = 1.12, 1.67) and SGA (OR = 1.25, 95% CI = 1.02, 1.52) than those in a DSM. The adjusted model for those who did not use ART showed no differences between infants born to parents in SSM and DSM (Table 4).

**Table 4:** Differences in birth outcomes between those born to same- and different-sex parents who did not use ART.

	SSM (N = 1,247) (%)	DSM (N = 308,719) (%)	Crude OR (95% CI)	Adjusted OR (95% CI)
PTB	9.5	7.1	1.37 (1.12, 1.67)	1.21 (0.98, 1.47)
SGA	9.5	7.7	1.25 (1.02, 1.52)	1.04 (0.85, 1.27)
LBW	4.8	3.8	1.26 (0.95, 1.64)	0.99 (0.74, 1.30)
LGA	7.3	7.7	0.95 (0.75, 1.18)	1.06 (0.84, 1.32)
Macrosomia	8.6	9.4	0.91 (0.73, 1.11)	1.03 (0.82, 1.26)

CI = confidence interval; DSM = different-sex marriage; LBW = low birth weight; LGA = large for gestational age; OR = odds ratio; SGA = small for gestational age; SSM = same-sex marriage.

## Discussion

Our study found that the prevalence of PTB was higher in infants born to SSM couples than DSM couples, even after adjusting for covariates. This difference was largely driven by those birthing parents who did not use fertility services. These findings are consistent with prior work showing worse perinatal outcomes among individuals who self-identified as lesbian or bisexual compared with heterosexual (1). This supports our hypothesis that minority stress may play a role in adverse birth outcomes for SSM couples. Interestingly, no differences were found for PTB among couples who used ART. However, ART use has been found to be associated with PTB (6, 7) and both SSM couples and DSM couples who used ART had higher proportions of preterm birth (SSM = 13.0%, DSM = 11.3%) than the couples who did not use ART (SSM = 9.5%, DSM = 7.1%). It is possible that the use of ART may be masking the existing disparities in PTB for SSM couples compared with DSM couples, although it is still possible that SSM couples who used ART experienced comparable infertility to DSM couples who used ART.

However, the present study differs from a similar study evaluating Massachusetts birth certificate data, in which the authors found no differences in perinatal outcomes (10). There are several factors that may explain the differences in SSM couples’ perinatal outcomes between the findings on the basis of Ohio data compared with the prior work with Massachusetts data. The SSM population giving birth in Ohio had lower income and less formal education than both the SSM population in Massachusetts and the DSM population in Ohio, and the use of WIC among SSM in Ohio was almost double that of those in Massachusetts (19% vs. 10%). These differences suggest that the SSM population in Massachusetts was far more resourced than the SSM population in Ohio, and likely able to access more supports and services during pregnancy. Additionally, the DSM couples in Massachusetts had a more diverse representation of racial/ethnic identities than in Ohio, but the racial/ethnic identity distribution among SSM couples was somewhat similar between the 2 states. It is possible that the intersection between sexual identity and racial/ethnic identity may play a role in some of the differences in findings.

Although all birthing parents in both Ohio and Massachusetts were in legally recognized marriages, SSM has been legally recognized in Ohio since 2015, whereas marriage equality in Massachusetts had been in place for 8 years before the study period. Further, Massachusetts has state-mandated comprehensive fertility coverage for commercial insurance, whereas Ohio does not (31). A higher proportion of Massachusetts SSM birthing parents used IVF compared with those in Ohio. Additionally, although Ohio does not have a commercial fertility mandate (31), 25% of SSM used ART compared with 2.6% of DSM. The specific reasons for using ART often differ between SSM and DSM. Although physiologic subfertility is typically a prerequisite for the use of ART in DSM couples, this is not the case for SSM couples. This highlights meaningful differences in their pathways to pregnancy, consistent with data in Massachusetts that found 34% of SSM used ART (8).

Significant differences in individual characteristics were found between those in SSM and DSM. Risk factors for fetal growth restriction occurred more among SSM (e.g., being older, nulliparous, more obese, smoking during pregnancy, lower education, lower socioeconomic status as indicated by receipt of public assistance) compared with DSM. The differences in risk factors associated with socioeconomic status may be of particular importance, considering previous research has supported that sexual minority individuals experience poorer social determinants of health related to socioeconomic status than heterosexual individuals (32). These risk factors suggest that this population may face more structural barriers to accessing medical care and to achieving healthy pregnancies.

Birthing persons in SSM in Ohio who used ART had a higher risk of macrosomia compared with DSM, even after controlling for parental body size. This finding may be explained by differences in the prevalence of polycystic ovary syndrome and diabetes across sexual orientation subgroups (33, 34).

The use of fertility-enhancing drugs or IVF was associated with PTB and SGA among DSM, but not among SSM. Many SSM couples conceive via ART without a diagnosis of infertility or subfertility. Current definitions of physiologic infertility do not apply as readily to those in SSM, and also may be harder to diagnose in most cases (35). For financial or logistical reasons, couples using donor sperm may sometimes use fertility medications without a diagnosis of infertility to theoretically shorten time to conception, despite research suggesting this does not increase ongoing pregnancy rates (36).

Further research is needed across different states to test whether these findings are generalizable to different regions and populations (e.g., populations with more racial/ethnic and socioeconomic diversity). Additionally, our data cannot be extrapolated to include the birth experiences of other sexual and gender minority populations (e.g., transgender and nonbinary people). Understanding the perinatal outcomes for all sexual and gender minority patients across the sexual orientation and gender spectrums is critical as we strive to improve their care. Future research on pregnancies in sexual and gender minority individuals should also examine underlying infertility and the use of ART as separate risk factors for fetal growth restriction.

### Strengths and limitations

This was among the first large-scale, representative studies of perinatal outcomes by sexual orientation and partner sex. This study’s strength is that it accounts for important covariates (37) (parental marital structure, child biological relatedness) by comparing pregnancies of birthing people in SSM with DSM. Our study also enabled the identification of our population of interest without sampling. Various measures of sexual orientation are used in research, and a lack of one consistent measure makes it difficult to compare findings across studies. With measures of sexual orientation, respondents must also be willing to disclose their sexual orientation; thus, sampling of sexual minorities runs the risk of selection bias that can change over time as the social climate changes (37). By using data from birth certificates, we were able to identify if participants were in SSM on the basis of reported gender without participants needing to disclose their sexual orientation or gender identity.

However, some limitations exist. Although we have reason to believe minority stress plays a role in pregnancy outcomes, our study design did not allow us to measure lived experiences of stigma nor compare variations in state policies related to discrimination. The intersection of heterosexism/homophobia and structural racism creates unique barriers for individuals with multiple marginalized identities during pregnancy, which can contribute to potentially worse perinatal outcomes for birthing parents who are multiply marginalized (5), but we were not able to measure this directly with the current study design. People in Ohio likely differ from those living elsewhere, so this study may not be generalizable to individuals in states with different fertility treatment coverage insurance policies or different levels of acceptance of same-sex couples. Additionally, this study was limited to individuals in SSM, and does not capture the experience of all sexual minority individuals in different-sex relationships, or unmarried people of all sexual orientations who may experience adverse reproductive outcomes (38). It would be worthwhile to examine perinatal outcomes among those who are not married as well to better understand whether marriage itself may be a determinant factor related to perinatal outcomes. Although a strength of this article was that individuals were not required to disclose their sexual orientation to be included, there may be important differences for bisexual, pansexual, and other sexual minority birthing parents in DSM that we were not able to identify. These populations experience minority stress but are largely understudied in reproductive health research. Thus, our findings do not generalize beyond birthing people in SSM (38). Similarly, transgender and nonbinary parents were likely included in our sample, yet given the limitations of birth certificate data, we were unable to determine parents’ gender identity. The measure used for this study asked for parent gender, but only allowed for parents to indicate their sex, likely resulting in misclassification of births to parents whose gender differs from their sex assigned at birth. Additionally, the use of birth certificate data did not allow us to examine the potential influence of the nonbirthing parent’s health, suggesting a potential avenue for future research. The use of birth certificate data also prevented us from being able to examine ART on a more granular level. For example, we were unable to distinguish between autologous IVF and reciprocal IVF, and birth outcomes may differ between those groups.

Finally, our study only observed live births, yet stressful events, like experiences of minority stress and stigmatization, may also increase the likelihood of miscarriage and stillbirth (39). Future studies would benefit from the inclusion of all pregnancies, including early pregnancy losses. These limitations are owed in part to the inherent complexity of sexual orientation identity, attractions, and behavior; research with sexual minority populations must engage with this complexity and ensure the research question is aligned with the interpretation of results (40).

Despite these limitations, our study adds to the limited evidence base on perinatal outcomes among sexual minorities and people in same-sex relationships.

## Conclusion

Our results provide evidence that people in SSM may face modestly increased risks for some perinatal outcomes, including PTB. Even in the face of increased exposure to minority stress, most people in SSM have healthy pregnancies and perinatal outcomes. The present findings have clinical implications for perinatal healthcare providers working with birthing people in same-sex partnerships and suggest that sexual minority birthing people may benefit from specialized care that takes into account the ways in which the unique lived experiences of people in same-sex partnerships may be associated with various perinatal outcomes.

## CRediT Authorship Contribution Statement

**Kendall A. Lawley:** Writing – review & editing, Writing – original draft, Visualization, Validation, Formal analysis. **Brittany M. Charlton:** Writing – review & editing, Writing – original draft. **Elizabeth Rubin:** Writing – review & editing, Writing – original draft. **Paula Amato:** Writing – review & editing, Writing – original draft. **Jae Corman:** Writing – review & editing, Writing – original draft, Validation, Supervision, Methodology, Conceptualization.

## Declaration of Interests

K.A.L. has nothing to disclose. B.M.C. has nothing to disclose. E.R. has nothing to disclose. Outside of the submitted work, P.A. reports a grant with Open Philanthropy, consulting fees from the World Health Organization, meeting/travel support from Oregon Health & Science University and the American Society for Reproductive Medicine, and is an American Society of Reproductive Medicine board member. J.C. has nothing to disclose.
